# Unusually Giant Sublingual Epidermoid Cyst: A Case Report

**Published:** 2016-07

**Authors:** Chintan-C Nishar, Vijayalaxmi-K. Ambulgekar, Atish-B. Gujrathi, Pravin-T. Chavan

**Affiliations:** 1 *Department of Otorhinolaryngology, Dr. Shankarrao Chavan Govt. Medical College, Nanded, India.*

**Keywords:** Epidermal Cyst, Intubation, Sublingual, Surgery, Tracheostomy

## Abstract

**Introduction:**

Epidermoid cysts are rare, slow‑growing, benign, developmental cysts, which are derived from abnormally situated ectodermal tissue. Epidermoid cysts of the floor of the mouth represent <0.01% of all oral cysts. So far, only a few cases have been reported.

**Case Report:**

Hereby, we present a case of a giant sublingual epidermoid cyst, which was completely asymptomatic upon presentation. However, due to its large size, it pushed the epiglottis posteriorly and created difficulty during intubation. The patient developed respiratory distress after its surgical excision and extubation, requiring tracheostomy post operatively. The patient recovered well and a successful weaning of tracheostomy was performed, giving the patient a healthy life.

**Conclusion:**

Epidermoid cyst is a rare differential diagnosis of sublingual swelling that should be kept in mind for large asymptomatic swellings in this region. The only symptom it can cause might be respiratory distress due to its large size. This can happen not only pre-operatively but also post-operatively and the surgeon should be ready for immediate tracheostomy.

## Introduction

Epidermoid cysts are rare, slow‑growing, benign, developmental cysts that are derived from abnormally situated ectodermal tissue. It is defined as, “a simple cyst lined with stratified squamous epithelium and lumen is filled with cystic fluid or keratin and no other specialized structure” (1). Epidermoid cysts seldom occur in the floor of the mouth though they can occur anywhere in the body. Most cases have been reported in the ovaries and the testicles (80%).The reported incidence of dermoid cysts of the head and neck region range from 1.6 to 6.9% and those located in the mouth make up less than 0.01% of all oral cysts (2-4). Epidermoid cysts develop from the entrapment of ectodermal elements at the fusion sites of the first and second branchial arches during the third and fourth embryonic weeks (5,6). Epidermoid cysts can occur at any age, from birth to 72 years; and they usually become apparent in patients between 15 and 35 years. Males are more commonly affected. It may present as small or large masses. It commences shortly after birth, grows slowly and painlessly. That is why it draws little attention until it causes discomfort (3,7,8). These cysts present either as solid or cystic masses in the midline of the neck between the suprasternal and submental region. They can occur lateral to the submandibular gland or rarely in the floor of the mouth. When large enough, they have the potential to cause symptoms of dysphagia and dyspnea with difficulty in speech. Here, we present a case of giant sublingual epidermoid cyst which was asymptomatic pre-operatively despite its huge size and long standing course. Yet it caused unexpected respiratory distress post-operatively, significant enough to warrant tracheostomy.

## Case Report

**Fig 1:**
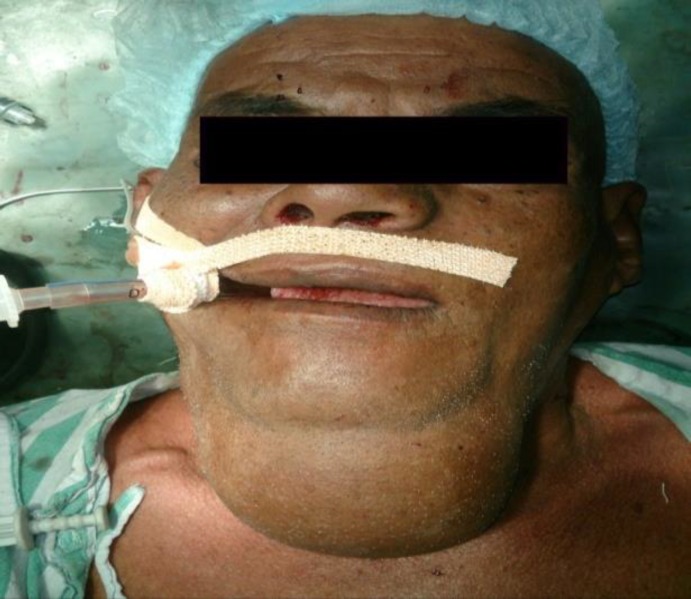
Preoperative front view

**Fig 2:**
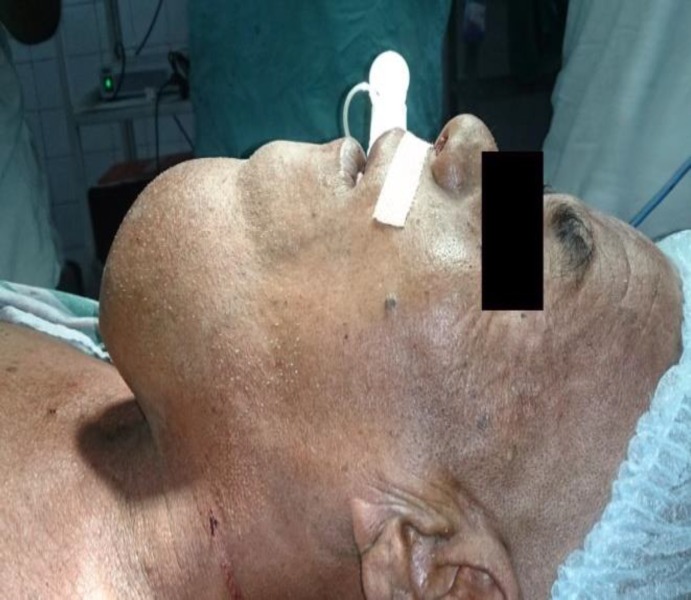
Preoperative side view

A 60 yr. old male presented to ENT OPD of our hospital with a large swelling in the submental region, which had been present for 30 yrs. Swelling was initially small but gradually increased over the years to reach its present size. The only complaint he had was a large visible swelling in the submental region. No other complaints like pain, dysphagia, dyspnea or difficulty in speech were associated with swelling. There was no history of sudden increase in size of swelling. Past medical, surgical, and dental history was also not relevant. During clinical examination, the swelling was found to be globular, around 13cm x 12cm in size, involving the whole submental region and extending to the right submandibular region (Figs.1,2).

Upon palpation, the swelling was soft to firm, with doughy consistency, non-tender, non-mobile, non-fluctuant, and non-trans illuminant. The swelling did not move with deglutition or with protrusion of tongue. Overlying skin was normal in color and texture and free from underlying swelling. Skin was easily pinchable. There was no sinus, discharge or ulcer seen. The swelling did not produce any significant bulge intra-orally in the floor of the mouth. 

However due to its large size, it caused significant posterior displacement of the epiglottis and hence indirect laryngoscopic examination could not be performed. With this clinical finding, differential diagnosis of epidermoid cyst, dermoid cyst or plunging ranula was made.

USG of the neck revealed a 10cm x 8cm well-defined, pseudo-solid cystic, space occupying lesion, containing dense homogenous internal echoes with few calcific specks giving acoustic shadow (mostly keratin). The lesion was pushing the right submandibular gland laterally.

**Fig 3:**
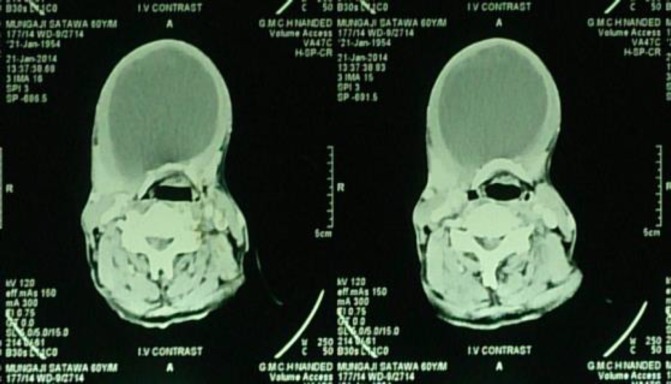
Preoperative CT scan

**Fig 4:**
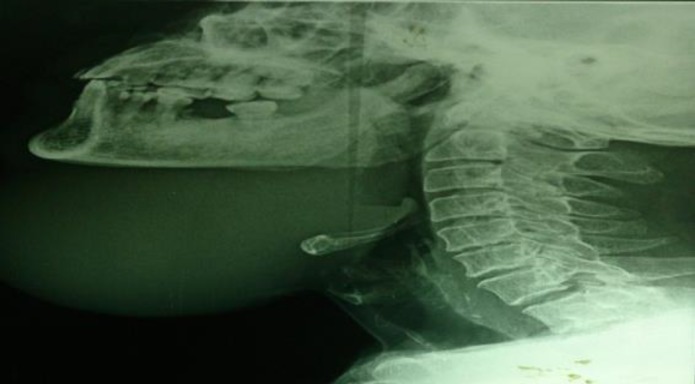
Preoperative X-ray

CT scan of the affected area showed a large, well-defined, thick walled, peripherally enhancing cystic lesion measuring 10.3cm x 8.1cm in the midline submandibular region (Figs. 3,4). 

**Fig 5:**
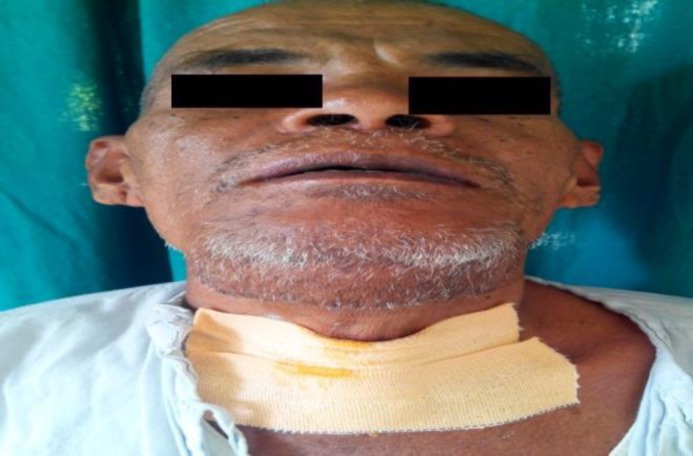
Postoperative front view

**Fig 6:**
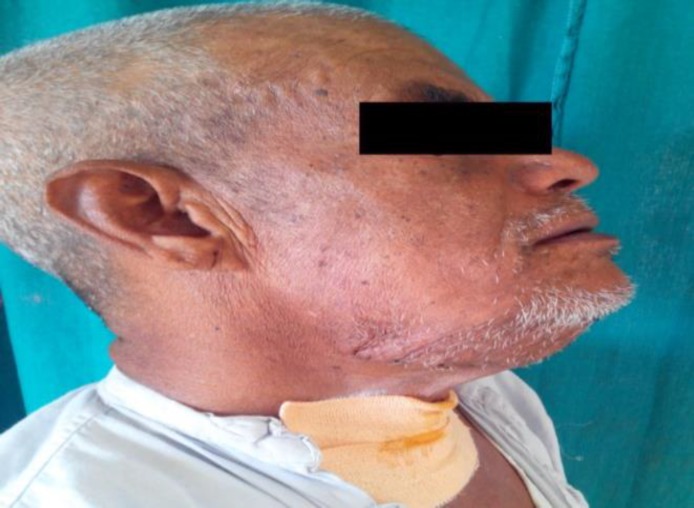
Postoperative side view

Swelling was separate from all surrounding structures. No enlarged lymph nodes were seen. No bony erosion was observed. Neck vessels were normal. Parotid, thyroid, and left submandibular gland were normal. FNAC from swelling suggested scantily cellular smear which showed anucleated squamous cells with scattered squamous epithelial cells on a background of keratinized material. All other laboratory investigations were within normal limits. Surgical excision of swelling was planned via an external approach under general anesthesia. However, due to excessively large swelling, the patient’s epiglottis was pushed posteriorly and routine intubation was not possible. He had to undergo fiber optic intubation and the airway was secured. After giving horizontal skin crease incision in the submental region, the yellow colored cyst wall was approached. It was completely separated from all surrounding structures and the cyst was carefully removed in toto by blunt dissection. The sample was sent for histopathological examination. Surgery was uneventful. However, during extubation, the patient developed respiratory distress and oxygen saturation fell, warranting immediate re-intubation. Later on, tracheostomy was performed to maintain patent airway. Adequate oxygen saturation was maintained with tracheostomy tube in situ and the patient recovered well. Sutures were removed on the 9^th^ post-op day. Tracheostomy weaning was done on the 10^th^ post-op day. The patient was discharged with a smiling face after successful weaning of tracheostomy and complete recovery (Figs. 5,6).

**Fig 7:**
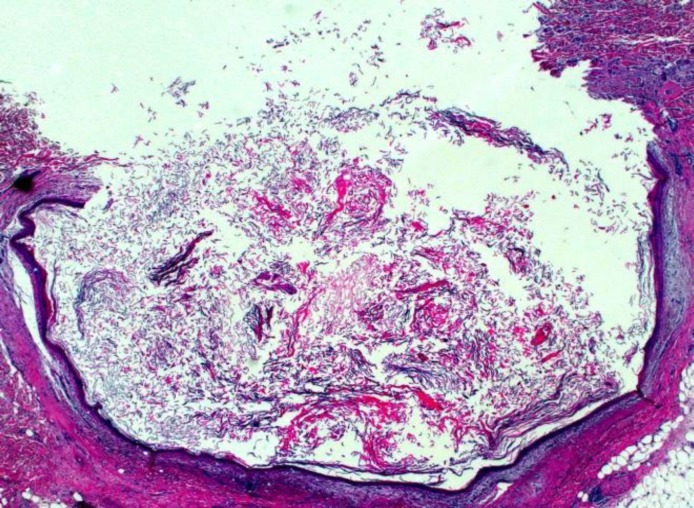
Histopathology of swelling

Histopathological examination revealed a thick walled cyst lined with a stratified squamous epithelium with acidophilic stratum corneum and basophilic dot like staining of stratum granulosum without any evidence of dermal appendages confirming the diagnosis of epidermoid cyst (Fig.7).

## Discussion

The differential diagnosis of swelling of the floor of the mouth and submental region includes-I: infection of the floor of the mouth involving sublingual or submandibular spaces, II: Ranula (plunging type), III: blockage of Wharton’s duct, IV: lipoma, V: thyroglossal duct cyst, VI: cystic hygroma, VII: branchial cleft cyst, VIII: benign and malignant tumor of the floor of the mouth and adjacent salivary gland (9). Absolute distinction between a simple Ranula, dermoid or epidermoid cysts cannot be made by radio imaging studies (10). Fine needle aspiration cytology (FNAC) helps in differentiating Ranula from dermoid or epidermoid. However, imaging studies especially magnetic resonance imaging (MRI) provides essential information of the cyst location allowing optimal surgical approach.

Epidermoid cysts are benign, ectoderm lined, inclusion cysts without any dermal appendages. Various theories have been proposed regarding the origin of the dermoid/ epidermoid cyst. As mentioned earlier, they may develop from the entrapment of epithelial remnants at the fusion site of the first and second branchial arches during the third to fourth fetal week. Some authors consider it as a variant of the thyroglossal duct cyst. Finally, previous surgical or accidental events may cause traumatic implantation of epithelial cells into deeper tissues which may give rise to an implantation dermoid (5,11). 

Epidermoid cysts may be categorized as congenital or acquired based on their origin, although there is no disparity between the two either clinically or histologically. They may be found in any age group, but show preponderance between 15 and 35 years of age with male predilection (12,13). In our case, the patient was a 60 yr. old male.

Clinically, epidermoid cysts are painless, slow growing lesion with doughy consistency, often soft and well encapsulated without any associated lymphadenopathy. They are usually not attached to any underlying structures and are completely free. They often remain asymptomatic for many years and grow painlessly. Usually attention is drawn when complications like masticatory or breathing difficulty arise as a result of a huge growth, as seen in this particular case. Large intraoral cysts usually present with dysphagia, dysphonia or dyspnea. Contents of the cyst can be keratinous, casseous, sebaceous or purulent (4). Clinicians must be cautious before needle aspiration or performing biopsy on the floor of the mouth because of its high risk of developing secondary infection and intensive inflammatory response due to high vascularity of the area (3). In our case, the cyst was not causing any symptoms of dysphagia or dyspnea preoperatively. It was not attached to any underlying structures like the hyoid bone, mandible or any neck vessel. 

Histologically, dermoid/epidermoid cysts are lined by stratified squamous epithelium with few rete processes.

Quite often, there is no granular cell layer and keratin from the surface of the epithelium can be seen to be sloughing into the cystic lumen, which is usually filled with degenerated and necrotic keratinaceous detritus. Areas of epithelial degeneration or ulceration may be seen, usually associated with a mild to moderately intense chronic inflammatory cell reaction. Inflammation may extend deeply into the sub-epithelial fibro-vascular stroma. In our case, the cystic lumen contained only keratin. No skin appendages in the cystic wall or any special structures like bone, muscle or odontogenic tissues were associated with it. Therefore, it was diagnosed as epidermoid cyst (9,14,15).

Endotracheal intubation in this kind of patient is also a challenging job. There is a high chance of rupture of the cystic lesion at the time of laryngoscopic examination, which may lead to aspiration of the cystic fluid. In addition, in our case, it was impossible to perform intubation in a regular laryngoscopic fashion as the epiglottis was pushed posteriorly; therefore, we had to perform fiber optic intubation which is a preferred procedure for such cases. The surgeon should keep the tracheostomy set ready to manage the airway in case of emergency to avoid catastrophe. The treatment of choice for epidermoid cysts is complete surgical excision. Recurrence is rare. Many surgical accesses have been described depending on the location and size of the lesion. Surgical approaches-such as transcutaneous, extended median glossotomy, median glossotomy and mid line incision, may be performed (11,16). Although rare, epidermoid cysts in sites other than the oral cavity harboring malignancy have been reported (17,18). However, no case of malignancy has been reported from the oral cavity epidermoids. In our case surgical procedure involved an extra oral approach because of the massive size of the lesion to enucleate it. Surgery was uneventful and the cyst was removed. However, the patient developed respiratory distress after extubation, probably due to a tracheal collapse.

Tracheostomy was performed and the airway secured. The patient recovered well and successful weaning of tracheostomy was performed later on. Throughout literature, epidermoid cysts of such a large size or such an incident have never been mentioned. This highlights a probable risk of such a large swelling causing respiratory distress even after successful removal of cyst. A surgeon must remain cautious until successful extubation of the patient and be sure that the patent’s airway is clear.

## Conclusion

Epidermoid cyst is rare occurrence in the head and neck region and more so in the oral cavity.

This diagnosis should be kept in mind when dealing with a large, painless, asymptomatic swelling in the sublingual region. Surgical excision is the treatment of choice for epidermoid cysts. All measures of securing the airway and tracheostomy must be kept ready while operating such a swelling, even after extubation.

## References

[1] Shafer WG, Hine MK, Levy BM (1993). A Text Book of Oral Pathology.

[2] Calderon S, Kaplan I (1993). Concomitant sublingual and submental epidermoid cysts: a case report. J Oral Maxillofac Surg..

[3] Cortezzi W, De Albuquerque EB (1994). Secondarily infected epidermoid cyst in the floor of the mouth causing a life threatening situation. J Oral Maxillofac Surg.

[4] Worley CM, Laskin DM (1993). Coincidental sublingual and submental epidermoid cyst. J Oral Maxillofac Surg.

[5] De Ponte FS, Brunelli A, Marchetti E, Bottini DJ (2002). Sublingual epidermoid cyst. J Craniofac Surg.

[6] Gold BD, Sheinkonf DE, Levy B (1974). Dermoid, Epidermoid and teratoid cysts of the tongue and the floor of the mouth. J Oral Surg.

[7] Yoshinari M, Nagayama M (1986). Epidermoid cyst of the uvula. J Oral Maxillofac Surg.

[8] Zachariades N, Skoura-Kafoussia C (1990). A life threatening epidermoid cyst of the floor of the mouth: report of a case. J Oral Maxillofac Surg.

[9] Howell CJT (1985). The sublingual dermoid cyst. Oral Surg Oral Med Oral Pathol.

[10] Coit WE, Harnsberger HR, Osborn AG, Smoker WR, Stevens MH, Lufkin RB (1987). Ranulas and their mimics: CT evaluation. Radiology.

[11] Longo F, Maremonti P, Mangone GM, De Maria G, Califano L (2003). Midline (dermoid) cysts of the floor of the mouth: report of 16 cases and review of surgical techniques. Plast Reconstr Surg.

[12] Damle MV, Irani DK, Hiranandani NL (2002). Epidermoid cyst of the floor of the mouth. Case report. Bombay Hosp J.

[13] Hemaraju N, Nanda SK, Medikeri SB (2004). Sub‑lingual dermoid cyst. Indian J Otolaryngol Head Neck Surg.

[14] Shear M (1992). Cyst of the oral regions.

[15] Walstad William R, Solomon James M, Schow Sterling R, Ochs Mark W (1998). Midline cystic lesion of the floor of the mouth. J Oral Maxillofacial Surg.

[16] Jham BC, Duraes GV, Jham AC, Santos CR (2007). Epidermoid cyst of the floor of the mouth: a case report. J Can Dent Assoc..

[17] Ikeda I, Ono T (1990). Basal cell carcinoma originating from an epidermoid cyst. J Dermatol.

[18] Lopez RF, Rodriguez Peralto JL, Castano E, Benito A (1999). Squamous cell carcinoma arising in a cutaneous epidermal cyst: Case report and literature review. Am J Dermatol Pathol.

